# miR-657 Targets SRCIN1 via the Slug Pathway to Promote NSCLC Tumor Growth and EMT Induction

**DOI:** 10.1155/2022/4842454

**Published:** 2022-08-17

**Authors:** Yingqian Zhang, Jiao Yuan, Mengfei Guo, Run Xiang, Xiang Wang, Tianpeng Xie, Xiang Zhuang, Qiang Li, Qi Lai

**Affiliations:** ^1^School of Basic Medical Science, Southwest Medical University, Luzhou 646000, China; ^2^Department of Respirology and Critical Care Medicine, Chengdu Seventh People's Hospital, Chengdu 610041, China; ^3^Department of Thoracic Surgery, Affiliated Traditional Medicine Hospital of Southwest Medical University, Luzhou 646000, China; ^4^Department of Thoracic Surgery, Sichuan Cancer Hospital & Institute, Sichuan Cancer Center, Cancer Hospital Affiliated to School of Medicine, UESTC, Chengdu 610000, China

## Abstract

**Background:**

MicroRNA- (miR-) 657 has been shown to regulate immunological and inflammatory activity, and it has also been defined to be dysregulated in both non-small-cell lung cancer (NSCLC) and hepatocellular carcinoma. The mechanistic role whereby miR-657 influences NSCLC progression, however, has yet to be clarified.

**Methods:**

miR-657 and SRCIN1 expression levels were assessed via qPCR in the cell lines and tissues of NSCLC. Besides, correlations between the levels of miR-657 and NSCLC patient pathological characteristics were examined, and the Kaplan-Meier approach was employed for the evaluation of the prognostic utility of miR-657 in these patients. Moreover, the Pearson correlation analyses and dual-luciferase reporter assessments were used for detecting interactive relationships between miR-657 and SRCIN1. In addition, CCK-8, EdU, and Transwell assessments were employed for the appraisal of the ability of miR-657/SRCIN1 to regulate NSCLC cell proliferation and invasion. Western blotting was employed for the assessment of the levels of NSCLC cell proteins associated with the epithelial-mesenchymal transition (EMT) that were influenced by miR-657. The nude mice xenograft tumor model is established to observe the effect of miR-657 on NSCLC growth *in vivo*.

**Results:**

NSCLC patient tissues and cell lines exhibited upregulated miR-657 expression that was closely related to tumor differentiation, lymphoid metastasis, and TNM stage. High levels of miR-657 were predictive of a poorer NSCLC patient prognosis, and overexpressing miR-657 resulted in the more rapid growth of NCI-H1650 and A549 cells, with a concomitant increase in their invasion. In addition, miR-657 overexpression raised the levels of Slug, N-cadherin, and Vimentin in these two cell lines while promoting E-cadherin downregulation. Dual-luciferase reporter assays confirmed that miR-657 was capable of binding to the SRCIN1 gene, and SRCIN1 expression levels were negatively associated with those of miR-657, indicating that it acts as a negative regulator of this gene. Knocking down SRCIN1 was capable to reverse the influences of miR-657 inhibitor treatment on NSCLC cell behavior. Finally, *in vivo* studies showed that miR-657 promoted NSCLC cell growth.

**Conclusion:**

The obtained findings illuminate that miR-657 can promote the growth of tumors and the induction of the EMT in NSCLC cells by targeting SRCIN1 expression and modulating Slug pathway activation, highlighting this pathway as a promising therapeutic target in cases suffering from NSCLC.

## 1. Introduction

One of the most prevalent forms of cancer is lung cancer, with correspondingly high rates of morbidity and mortality. Over 85% of the cases of lung cancer are stratified under the non-small-cell lung cancer (NSCLC) subtype [1, 2]. While there have been substantial advances in preclinical and clinical research aimed at treating NSCLC, patient outcomes remain relatively undesirable, with a 5-year rate of survival for just 15% following diagnosis [3].

MicroRNAs (miRNAs) are small RNAs that lack coding potential yet can bind to complementary regions within the 3′-untranslated regions (UTRs) of target mRNAs to control their expressions at the posttranscriptional level [4, 5]. Studies have clearly demonstrated that miRNAs can regulate cellular processes closely tied to tumorigenesis including proliferation, cell cycle induction, apoptosis, metastasis, and invasion [6]. Consistently, many miRNAs have been represented to be dysregulated in the context of cancer, with specific miRNAs acting to promote or inhibit cancer development [7, 8]. Recent research has shown miR-657 to function as a regulator of immunological and inflammatory processes that can regulate insulin growth factor 2 receptor (IGF2R) expression in type 2 diabetes [9]. In placental tissues, miR-657 dysregulation has been detected and associated with the incidence of gestational diabetes owing to its ability to regulate local inflammatory activity [10]. There is also information that this miRNA performs a task in oncogenic settings, with its differential expression having been reported in NSCLC and hepatocellular carcinoma [11, 12]. The specific mechanisms whereby miR-657 can influence NSCLC progression, however, have yet to be clarified.

SRC Kinase Signaling Inhibitor 1 (SRCIN1) functions as a regulator that is capable of inhibiting the migration and spreading of cells, as well as regulating Ca-dependent exocytosis. In breast cancer, lung cancer, and osteosarcoma, SRCIN1 has been demonstrated to function as a tumor suppressor [13, 14]. Wang et al., for example, determined that SRCIN1 was able to enhance *in vitro* E-cadherin expression and to thereby suppress osteosarcoma cell growth [15], while Sun et al. ascertained that miR-181a was capable to suppress SRCIN1 expression in colorectal cancer cells, thereby modulating SRC/VEGF signaling to influence angiogenic activity [16]. Here, the bioinformatics analysis found that SRCIN1 is a potential target gene of miR-657. Despite there have been multiple published studies examining the functional importance of SRCIN1, miR-657 mediates SRCIN1 regulation in the context of tumorigenesis which is still incompletely understood, underscoring the need for additional studies of the function of this gene as a regulator of NSCLC development and progression.

Herein, we found miR-657 to be upregulated in both NSCLC patient tumors and cell lines relative to paired control tissue samples or normal pulmonary epithelial cells. Mechanistically, this miRNA was found to enhance the proliferation, invasive activity, and EMT induction in the cells of NSCLC. Furthermore, SRCIN1 was identified as a straightforward miR-657 target gene that was able to inhibit Slug activation within NSCLC cells, thereby regulating malignant activity. Together, these data offer novel insights into NSCLC pathogenesis and highlight an important pathway that may offer value as a biomarker for the future treatment and/or diagnosis of NSCLC.

## 2. Materials and Methods

### 2.1. Sample Collection

In total, NSCLC tumors and paired paracancerous tissues were harvested from 96 newly diagnosed patients with NSCLC undergoing surgery from July 2014 to March 2016 at the Affiliated Traditional Medicine Hospital of Southwest Medical University and stored for further analysis. None of the NSCLC patients were received radiation or chemotherapy prior to surgery. In addition, clinical findings and follow-up data pertaining to these patients were recorded. Furthermore, sixty-seven NSCLC patients' blood and forty-sixth healthy volunteers' blood were collected for detecting the circulating miR-657 expression in the blood samples from August 2020 to November 2021 at Sichuan Cancer Hospital & Institute, Sichuan Cancer Center. The patient's informed consents have been procured from all volunteers before experiments, and the Ethics Committee of the Affiliated Traditional Medicine Hospital of Southwest Medical University confirmed this study (201903-9).

### 2.2. Cell Culture and Transfection

The NCI-H1650, NCI-H358, A549, NCI-H1299, and HCC827 NSCLC cell lines and the control BEAS-2B cell line were acquired from the Cell Bank (Shanghai, China) and cultivated in DMEM supplied with 10% FBS (Gibco, MD, USA). Media was exchanged every other day, and cells were passaged (1 : 3) every 6 days. miRNA mimics are RNAs to mimic the endogenous miRNAs, which are synthesized by chemical synthesis and transfected into cells to enhance the function of endogenous miRNAs. miRNA inhibitors are chemically modified inhibitors that target specific miRNAs in cells. Mimic NC and inhibitor NC are their negative controls. Here, miR-657 mimic, inhibitor, and corresponding negative control (NC) constructs were from RiboBio (Guangzhou, China), while an SRCIN1-specific shRNA (5′-AATTCAAAAAAAGCCAAACTACTGGAGTTTCAAGTCTCTTGAAGTAGCACAACATTCTCCACCCG-3′) and a control shRNA (5′-AATTCAAAAAATTCTCCGAACGTGTCACGTTCTCTTGAATCAGAAAGTTGCTCTTCAGCCG-3′) were acquired from GenePharma (Shanghai, China). Overexpression was achieved by cloning the SRCIN1 coding sequence into the pcDNA3.0 vector. All cells were transfected with appropriate constructs via Lipofectamine 2000 (Invitrogen, CA, USA) based on provided directions when 80-90% confluence. At 6 h posttransfection, the milieu was substituted with a fresh complete milieu, and cells were collected for next utilization at 24-48 h posttransfection.

### 2.3. CCK-8 Assay

Cells were added to the plates containing 96 wells (2 × 10^3^/well), with absorbance at 450 nm being measured at appropriate time points with a CCK-8 kit (Dojindo Laboratories, Kumamoto, Japan) for the assessment of cell viability.

### 2.4. Fluorescence *In Situ* Hybridization

A kit of Fluorescent In Situ Hybridization (FISH) (RiboBio, Guangzhou, China) was implemented on the basis of the presented directions. In brief, cells were rinsed with PBS, fixed for 10 min employing 4% formaldehyde, permeabilized for 5 min at 4°C using 0.5% Triton X-100, rinsed thrice with PBS (5 min/wash), prehybridized for 30 min at 37°C, and probed overnight with an anti-miR-657 oligodeoxynucleotide probe in hybridization solution at 37°C while protected from illumination. DAPI was then used for counterstaining, and cells were imaged with a confocal laser-scanning microscope (Carl Zeiss). The fluorescence intensity was quantified using ImageJ (V 1.8.0, National Institutes of Health, USA).

### 2.5. Immunohistochemical Staining

Initially, NSCLC tumors and paracancerous tissues were fixed with 4% paraformaldehyde for 24 h, after which they were subjected to immunohistochemical (IHC) staining for SRCIN1 using primary anti-SRCIN1 (abcam, ab5407; 1 : 200), and secondary antibody HRP Donkey Anti-Rabbit IgG (H+L) (ABclonal, AS038, 1 : 1000). Samples were then imaged via light microscope (Olympus, Tokyo, Japan).

### 2.6. Immunofluorescence (IF)

Cells were cultured in 24-well plates on coverslips for 24 hours, then coverslips were collected, and cells were fixed with 4% formaldehyde and permeabilized with 0.5% Triton X-100 at room temperature. Followed, cells were incubated with polyclonal anti-E-cadherin (abcam, ab231303, 1 : 300) and anti-N-cadherin (abcam, ab98952, 1 : 300), and then incubation with Cy3 or FITC-labeled secondary antibody (ABclonal, Wuhan, China). Cells were then stained with DAPI (Beyotime, Shanghai, China) for 5 min. Images were captured under a fluorescent microscope.

### 2.7. qPCR

TRIzol (Invitrogen, CA, USA) was employed for the extraction of RNA, followed by cDNA preparation and use in qPCR analyses conducted via the SYBR Green method (TaKaRa, Otsu, Shiga, Japan). All first-strand cDNA preparation for miRNAs was conducted via the stem-loop method [17] with a cDNA Synthesis Kit (R601, Novabio, Shanghai, China). Stem-loop primers (5′-CTCAACTGGTGTCGTGGAGTCGGCAATTCAGTTGAGCCTAGAGA-3′) were obtained from General Biol (Anhui, China). Thermocycler settings for qPCR were as follows: 40 cycles of 94°C for 30 s, 55°C for 30 s, and 72°C for 90 s. The 2^-*ΔΔ*Ct^ technique was implemented for the evaluation of the relative gene expression. The primer sequences were listed as follows: miR-657: F: 5′-ACACTCCAGCTGGGGGCAGGTTCTCACCC-3′ and R: 5′-CTCAACTGGTGTCGTGGA-3′ and SRCIN1: F: 5′-GAGGCTCGCAACGTCTTCTAC-3′ and R: 5′-GCGATGCGTACACCATCTCTC-3′. GAPDH was served as the internal control for SRCIN1. U6 was the internal control of miR-657. Primers for U6 were as follows: forward, 5′-CTCGCTTCGGCAGCACA-3′and reverse, 5′-AACGCTTCACGAATTTGCGT-3′. Primers for GAPDH were as follows: forward, 5′-CTGGGCTACACTGAGCACC-3′and reverse, 5′-AAGTGGTCGTTGAGGGCAATG-3′.

### 2.8. EdU Assay

Cellular proliferation was quantified with an EdU (5-ethynyl-20-deoxyuridine) assay kit (RiboBio, Guangzhou, China). Cells were then added to confocal plates (10 × 10^5^/well), followed by incubation for 2 h at 37°C with 50 *μ*M of EdU buffer. Cells were then fixed by employing 4% formaldehyde for 30 min and permeabilized for 20 min with 0.1% Triton X-100, and EdU solution was added to culture media. Besides, for nuclear counterstaining, Hoechst was implemented, and cells were imaged by applying a fluorescence microscope.

### 2.9. Transwell Assays

Cells were suspended at 3 × 10^4^ cells/ml, and 100 *μ*l of cells was added into the upper chamber of a Matrigel-coated Transwell insert (Corning, NY, USA). Then, 600 *μ*l of media supplied with 20% FBS was added to the lower chamber. The incubation of cells was executed for 24 h, following which migratory cells were fixed for 15 min using methanol, stained for 20 min using crystal violet, and counted via microscopy. Cells in 5 haphazard fields of view were then calculated (200x).

### 2.10. Western Blotting

Radioimmunoprecipitation assay (RIPA) was utilized to extract total cellular protein content, after which a BCA assay (Beyotime, Shanghai, China) was used for protein quantification. Samples were then electrophoretically separated and transferred onto PVDF membranes (Millipore, MA, USA). Following blocking for 2 h with 5% nonfat milk, blots were examined with proper primary and secondary antibodies. The bands of protein were then discovered via enhanced chemiluminescence (ECL) and analyzed with ImageJ (NIH, MD, USA). Antibodies used in this study were as follows: E-cadherin (abcam, ab231303, 1 : 1000), N-cadherin (abcam, ab98952, 1 : 1000), Vimentin (abcam, ab92547, 1 : 1000), Slug (abcam, ab27568, 1 : 1000), Snail (abcam, ab216347, 1 : 1000), HRP Goat Anti-Rabbit IgG (H+L) (ABclonal, AS014, 1 : 4000), HRP Goat Anti-Mouse IgG (H+L) (ABclonal, AS003, 1 : 5000).

### 2.11. Dual-Luciferase Reporter Assay

The TargetScan database (https://www.targetscan.org/vert_80/) was used to analyze the binding sites between miR-657 and SRCIN1. SRCIN1-wide type (WT) and SRCIN1-mutated type (MUT) vectors were all constructed by GenScript (Nanjing, China). Lipofectamine 2000 was employed to cotransfect cells with miR-NC/miR-657 mimic and SRCIN1-WT/SRCIN1-MT into 293T cells. At 48 h posttransfection, cells were lysed, and for the measurement of luciferase activity, a dual-luciferase reporter assay system (Promega, WI, USA) was implemented.

### 2.12. RNA Immunoprecipitation

RIP assessments were exerted through employing a Magna RIP™ RNA-Binding Protein Immunoprecipitation Kit (Millipore) based on provided directions. The specificity of binding interactions was assessed using species-matched control IgG (*n* = 3). AGO2 is a key protein of RNA-induced silencing complex (RISC), and these guide miRNAs direct RISC to complementary mRNAs that are targets for RISC-mediated gene silencing. For anti-AGO2 RIP analyses, miRNA mimics were transfected into A549 cells (using 2 *μ*g/ml of anti-AGO2), and RIP cleavage was conducted at 48 h posttransfection.

### 2.13. RNA Pull-Down

The RNA pull-down assay was performed as previously reported [18]. Briefly, 100 nM biotinylated or nonbiotinylated miR-657 and 100 nM biotinylated or nonbiotinylated antisense of miR-657 (GenePharma, Shanghai, China) were transfected into A549 cells by Lipofectamine™ 3000 reagent. After 24 h, 1 × 10^7^ cells were collected and lysed in the lysis buffer, and 500 *μ*l of cell lysate was incubated with 500 *μ*l of washed streptavidin magnetic beads (Life Technologies, USA) for 2 h at 37°C. The beads were washed with wash buffer for three times, and RNA was extracted with TRIzol reagent. The coprecipitated RNA was analyzed using PCR.

### 2.14. Tumor Xenograft Models

All animal experiments were done in animal laboratory center as per the study protocol according to the NIH Guide for the Care and Use of Laboratory Animals, approved by the Animal Care and Use Committee of Sichuan Cancer Hospital & Institute, Sichuan Cancer Center. Nude BALB/c mice (4 weeks old, female) were obtained from Charles River (Beijing, China). The mice were housed under pathogen-free conditions and were randomly assigned to five groups of six mice each. 0.5 × 10^7^ of transfected A549 cells were injected subcutaneously into the left flanks. After 3 days, the xenografts were injected with micrON hsa-miR-657 agomir (2.5 nmol, RiboBio, Guangzhou, China) or micrOFF hsa-miR-657 antagomir (2.5 nmol, RiboBio, Guangzhou, China) twice a week. Tumor volumes were evaluated weekly for four weeks, with volumes measured with calipers and determined as *V* = length × width^2^ × 0.5. The mice were sacrificed after four weeks and the tumors were extracted for further study.

### 2.15. Statistical Analysis

SPSS 19.0 (IBM Corp., NY, USA) and GraphPad Prism 7.0 (CA, USA) were implemented to analyze all outcomes. Results were given as means ± standard deviation and were scrutinized with the aid of Student's *t*-tests. The curves of the Kaplan-Meier and log-rank assessments were employed to evaluate differences in survival outcomes between groups, while associations between miR-657 and SRCIN1 were appraised through the Pearson correlation assessments. *P* < 0.05 was the significance threshold.

## 3. Results

### 3.1. NSCLC Tumor Tissues Exhibit miR-657 Upregulation That Is Predictive of Poor Prognostic Outcomes

**Figure 1:**
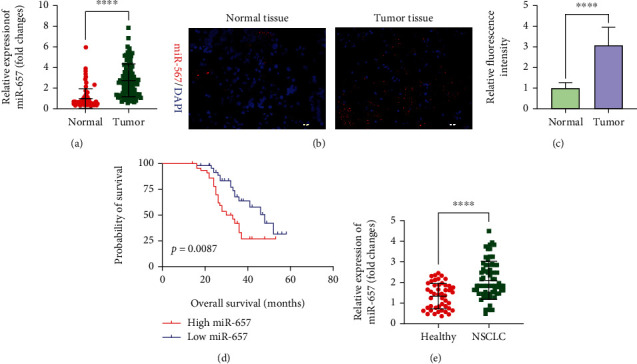
NSCLC tissues exhibit miR-657 upregulation that is predictive of poor prognostic outcomes. (a) miR-657 expression in NSCLC patient tumors and normal (paracancerous) tissues was measured via qPCR, with a normal tissue being used for normalization, *n* = 96. (b) miR-657 levels in tissues were measured via FISH. (c) Quantification of the FISH results. (d) The relationship between overall survival and miR-657 expression was examined using the Kaplan-Meier curves. (e) The circulating miR-657 expressions in the blood of NSCLC patients (*n* = 67) were significantly increased compared with healthy volunteers (*n* = 46). Data are means ± standard deviation from three replicate assessments. ^∗∗∗∗^*P* < 0.0001.

We began by analyzing miR-657 expression in 96 paired NSCLC patient tumors and paracancerous tissue samples via qPCR and FISH, revealing the marked upregulation of this miRNA in NSCLC tumors (Figures 1(a)–1(c)). Moreover, miR-657 was found to be correlated with NSCLC patient TNM staging, lymph node metastasis, and tumor differentiation (Table 1). Subsequently, cases were stratified into two groups according to median (cutoff value = 2.588) of miR-657 expression levels, with overall survival outcomes then being compared between these groups via the Kaplan-Meier approach, demonstrating that higher levels of miR-657 expression were closely tied with poorer patient survival as compared to lower levels of expression for this miRNA (Figure 1(d)). Furthermore, we detected the circulating miR-657 expression in the blood of NSCLC patients, and we found that the expression levels of miR-657 in NSCLC patients' blood (*n* = 67) were significantly increased compared with healthy volunteers (*n* = 46) (Figure 1(e)). As such, the obtained outcomes illuminate that miR-657 may play important roles in the progression of NSCLC.

### 3.2. miR-657 Promotes EMT Induction to Enhance the Proliferation and Invasiveness of NSCLC Cell Lines

**Figure 2:**
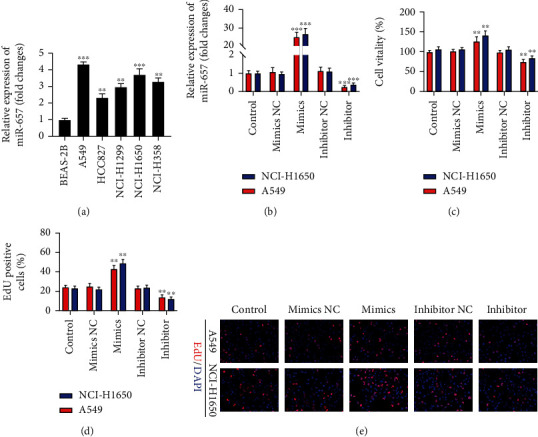
miR-657 enhances proliferative activity in NSCLC cell lines. (a) qPCR was employed for the appraisal of miR-657 expression in the cell lines of NSCLC and BEAS-2B cells, with control values being used for normalization. (b) qPCR was used to confirm the successful transfection of NCI-H1650 and A549 cells with miR-657 mimics and inhibitors. (c) The viability of NCI-H1650 and A549 cells following miR-657 inhibitor, mimic, or NC transfection was measured. (d and e) The proliferation of H460 and A549 cells transfected as indicated was assessed via EdU assay. Data are means ± standard deviation from three replicate assessments. ^∗^*P* < 0.05, ^∗∗^*P* < 0.01, and ^∗∗∗^*P* < 0.001.

To discover the functional importance of miR-657 in NSCLC, A549 and NCI-H1650 cells were next utilized in gain- and loss-of-function assessments aimed at evaluating its impact on proliferation, invasion, and EMT induction. Initially, miR-657 expression was assessed via qPCR in six NSCLC cell lines, revealing that it was substantially upregulated in HCC827, NCI-H1650, NCI-H358, A549, and NCI-H1299 cells as compared with control normal pulmonary epithelial (BEAS-2B) cells (Figure 2(a)); according to the results, two cell lines A549 and NCI-H1650 had the highest expression of miR-657, which were selected for subsequent studies. Next, the effectiveness of miR-657 mimic or inhibitor transfection in A549 and NCI-H1650 cells was assessed (Figure 2(b)). When suppression of miR-657 impaired the viability of NSCLC cell lines, whereas its overexpression resulted in the opposite effect (Figure 2(c)). An EdU uptake assay further revealed a marked increase in NSCLC cell proliferation following miR-657 mimic transfection relative to mimic NC transfection, whereas miR-657 inhibitor transfection markedly suppressed such proliferative activity relative to appropriate inhibitor NC transfection (Figures 2(d) and 2(e)).

**Figure 3:**
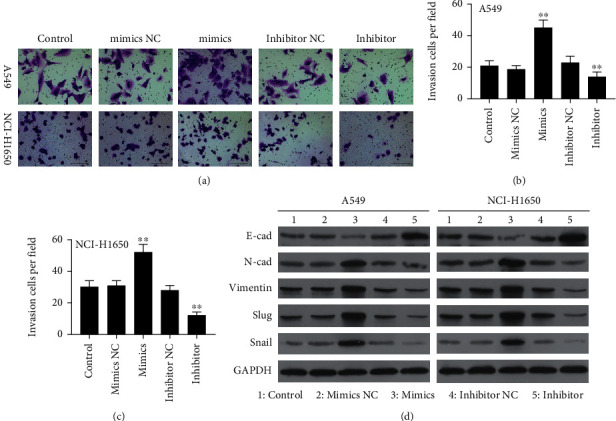
miR-657 upregulates EMT marker expression and promotes increased invasiveness in NSCLC cells. (a–c) The invasiveness of NCI-H1650 and A549 cells transfected with miR-657 mimic, inhibitor, or correspondent NC control constructs was assessed using Transwell invasion assay (200x). (d) Western blotting was employed for the evaluation of N-cadherin, E-cadherin, Vimentin, Slug, and Snail protein levels in A549 and NCI-H1650 cells transfected as indicated. Outcomes are means ± standard deviation from three replicate assessments. ^∗∗^*P* < 0.01.

The invasiveness of both A549 and NCI-H1650 cells was promoted following miR657 mimic transfection, while miR-657 inhibitor transfection had the opposite effect (Figures 3(a)–3(c)). Western blotting was additionally utilized to assess EMT-associated protein expression, revealing that miR-657 mimic transfection suppressed E-cadherin levels and enhanced N-cadherin, Vimentin, Slug, and Snail expressions within NSCLC cells. On the contrary, the opposite effect was detected after miR-657 inhibitor transfection (Figure 3(d)). These data thus suggest that miR-657 can promote NSCLC cell invasion and proliferation in part via driving EMT induction *in vitro.*

### 3.3. miR-657 Directly Targets SRCIN1

**Figure 4:**
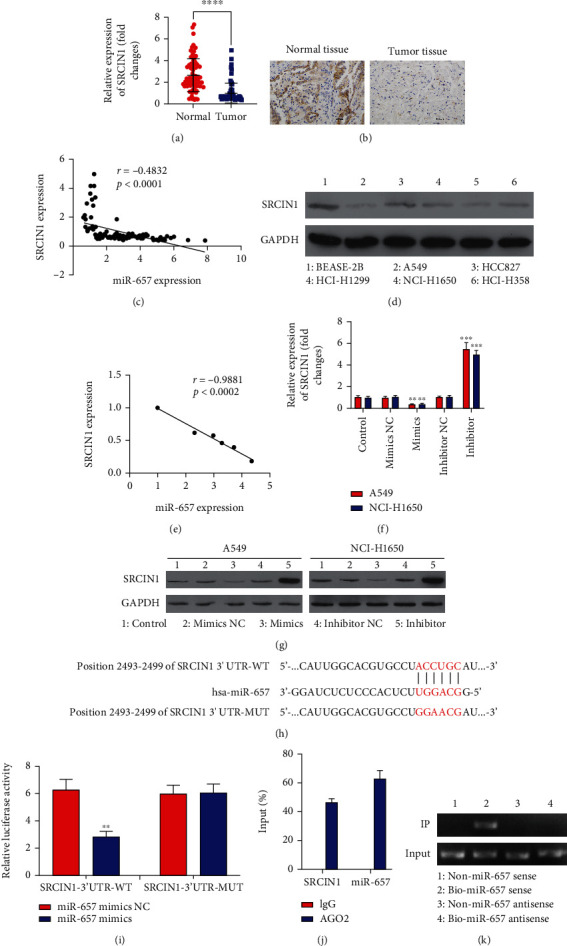
miR-657 directly targets SRCIN1. (a) SRCIN1 mRNA levels in NSCLC tumors and normal tissues were measured through qPCR (*n* = 96). (b) IHC staining was employed to assess SRCIN1 expression in 96 NSCLC patient tumor and healthy control tissue samples, with representative images being shown (×200, scale bar 100 *μ*m). (c) SRCIN1 and miR-657 expression levels were negatively correlated. (d) SRCIN1 levels were measured in different cell lines. (e) SRCIN1 and miR-657 expression levels were negatively correlated in different cell lines. (f) SRCIN1 levels were measured in the indicated NSCLC cell lines following miR-657 mimic, inhibitor, or corresponding NC construct transfection. (g) SRCIN1 protein levels were assessed in NSCLC cell transfected as indicated. (h) SRCIN1 and miR-657 sequence complementarity. (i) Luciferase activity levels were appraised in 293T cells after miR-NC/miR-657 mimic or SRCIN1-WT/SRCIN1-MT cotransfection. (j) RIP assessments were employed to appraise the ability of miR-657 and SRCIN1 to interact in A549 cells. (k) RNA pull-down results showed that miR-657 could band with SRCIN1 in A549 cell lines. Data are means ± standard deviation. ^∗∗^*P* < 0.01 and ^∗∗∗^*P* < 0.001.

Next, we assessed SRCIN1 expression in NSCLC tissue samples, revealing it to be downregulated in tumors in relation to paracancerous tissues (Figures 4(a) and 4(b)). In the Pearson correlation assessments, SRCIN1 and miR-657 expression levels were negatively correlated in NSCLC tissue samples (Figure 4(c)). Furthermore, we also analyzed the protein expression of SRCIN1 in different cell lines, we found SRCIN1 protein was decreased in NSCLC cell lines (Figure 4(d)), and its expression levels were negatively correlated with miR-657 expression levels (Figure 4(e)). Transfecting miR-657 mimics into both A549 and NCI-H1650 cells led to SRCIN1 downregulation (Figures 4(f) and 4(g)). Moreover, the predictive TargetScan algorithm determined SRCIN1 as a direct miR-657 target gene (Figure 4(h)), and luciferase assays confirmed this regulatory relationship as evidenced by a marked drop in luciferase activity following miR-657 mimic and SRCIN1-WT cotransfection in 293T cells (Figure 4(i)). Moreover, RIP assays additionally indicated that SRCIN1 and miR-657 coprecipitated together, again validating the predicted interaction between miR-657 and the SRCIN1 3′-UTR in A549 cell line (Figure 4(j)). RNA pull-down results showed that miR-657 could band with SRCIN1 in A549 cell line (Figure 4(k)). These results thus confirmed the identity of SRCIN1 as a direct miR-657 target gene that was negatively related to the expression of this miRNA in NSCLC.

### 3.4. SRCIN1 Inhibits NSCLC Cell Invasion, Proliferation, and EMT Induction

**Figure 5:**
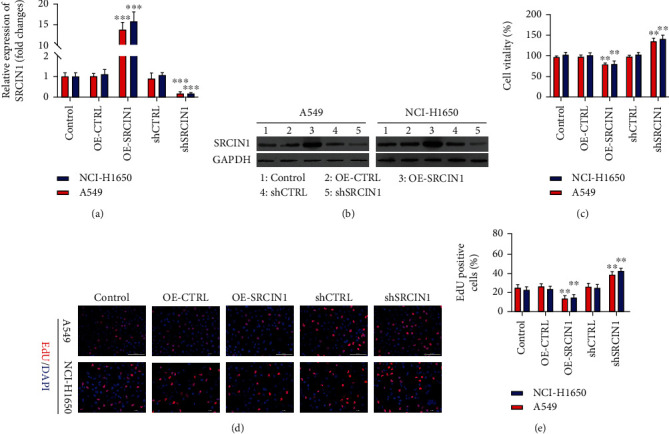
Overexpression of SRCIN1 suppresses NSCLC cell proliferation. NSCLC cells were transfected as indicated to overexpress or knock down SRCIN1. (a and b) SRCIN1 expression was appraised through qPCR and Western blotting after the knockdown or overexpression of SRCIN1. (c) The proliferation of NSCLC cell lines transfected as indicated was measured via CCK-8 assessment. (d and e) The proliferation of NCI-H1650 and A549 cells was measured via EdU assay. Data are means ± standard deviation. ^∗^*P* < 0.05, ^∗∗^*P* < 0.01, and ^∗∗∗^*P* < 0.001.

SRCIN1 expression levels were next modulated in A549 and NCI-H1650 cells in an effort to establish the task of this gene in the regulation of NSCLC cell proliferative, invasive, and EMT induction activity. qPCR and Western blotting confirmed the successful knockdown and overexpression of SRCIN1 following shSRCIN1 and OE-SRCIN1 plasmid transfection, respectively (Figures 5(a) and 5(b)). CCK-8 assays revealed at 48 h posttransfection, SRCIN1 overexpression suppressed the proliferation of both NCI-H1650 and A549 cells (Figure 5(c)). Consistently, an EdU uptake assay revealed that SRCIN1 overexpression repressed the capability of HCC827 and H1299 cells to proliferate (Figures 5(d) and 5(e)).

**Figure 6:**
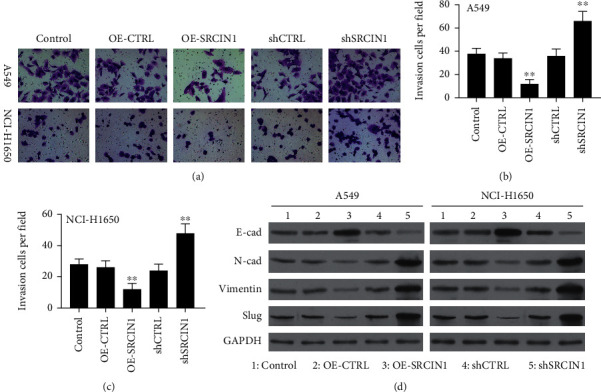
SRCIN1 inhibits NSCLC cell invasion and EMT induction. SRCIN1 was knocked down or overexpressed in A549 and NCI-H1650 cells. (a–c) SRCIN1 inhibited NSCLC cell invasion, as determined by plating 2 × 10^4^ cells treated as appropriate to the upper chamber of a Transwell insert, removing noninvasive cells after an appropriate period of time, and imaging the remaining cells (100x). Representative images are shown with corresponding quantification. (d) SRCIN1 suppressed the expression of EMT marker proteins in the indicated NSCLC cell lines, with GAPDH serving as a loading control. Outcomes are given as mean ± standard deviation, *n* = 3. ^∗∗^*P* < 0.01.

Next, Transwell assessments were employed to appraise the invasiveness of these cell lines, revealing that SRCIN1 overexpression suppressed both A549 and NCI-H1650 cell invasion *in vitro* (Figures 6(a)–6(c)). Western blotting additionally demonstrated that SRCIN1 overexpression resulted in increases in E-cadherin expressions and reductions in Slug, Vimentin, and N-cadherin expressions, whereas the opposite changes were detected when SRCIN1 was knocked down in these cells (Figure 6(d)). Conclusively, the obtained outcomes illuminate that SRCIN1 functions as an inhibitor of NSCLC cell invasion, proliferation, and EMT induction.

### 3.5. miR-657 Regulates NSCLC Cell Malignancy in an SRCIN1-Dependent Manner

**Figure 7:**
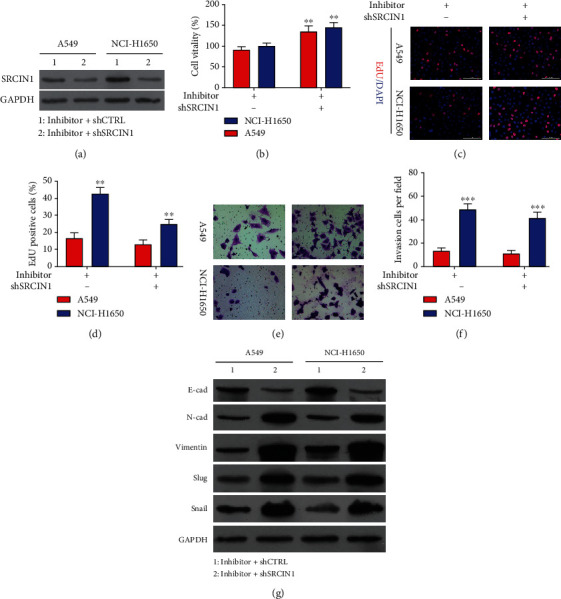
miR-657 regulates NSCLC cell malignancy in an SRCIN1-dependent manner. A549 and NCI-H1650 cells were transfected with a miR-657 inhibitor with or without shSRCIN1. (a) Western blotting was used for measuring SRCIN1 protein expressions in the indicated cell lines. (b) NCI-H1650 and A549 cell viability was appraised at 24, 48, and 72 h. (c and d) The proliferation of NCI-H1650 and A549 cells was appraised via EdU assessment. (e and f) The invasion of A549 and NCI-H1650 cells was assessed in a Transwell assessment. (g) Western blotting was employed to measure N-cadherin, E-cadherin, Vimentin, and Slug protein levels in NSCLC cell lines transfected as indicated, with GAPDH serving as a loading control. Outcomes are means ± standard deviation. ^∗∗^*P* < 0.01 and ^∗∗∗^*P* < 0.001.

To more fully clarify the relationship between SRCIN1 and miR-657, rescue experiments were next performed in which SRCIN1 was knocked down in A549 and NCI-H1650 cells following miR-657 inhibitor transfection to reverse the upregulation of this gene (Figure 7(a)). Such shSRCIN1 cotransfection was sufficient to partially reverse the observed reductions in NSCLC cell viability following miR-657 inhibitor transfection (Figure 7(b)), and EdU uptake assays similarly indicated that miR-657 inhibitors impaired the proliferation of both A549 and NCI-H1650 cells, while SRCIN1 knockdown blunted this effect (Figures 7(c) and 7(d)). Consistently, SRCIN1 knockdown reversed the adverse influence of miR-657 inhibitor transfection on NSCLC cell invasion (Figures 7(e) and 7(f)). SRCIN1 silencing also attenuated miR-657 inhibitor-dependent E-cadherin upregulation and Slug, Vimentin, and N-cadherin downregulation (Figure 7(g)). These data all above suggest that downregulating SRCIN1 was sufficient to complete reverse the regulatory influence of miR-657 inhibitor treatment on NSCLC cell proliferation, invasion, and also EMT induction.

### 3.6. miR-657 Promotes Tumor Growth *In Vivo*

**Figure 8:**
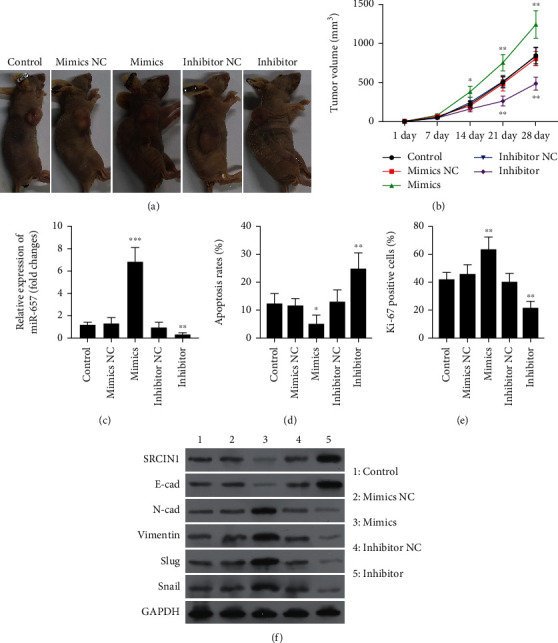
miR-657 promotes tumor growth *in vivo*. (a and b) miR-657 overexpression tumors were significantly larger than those of control mice, while the miR-657 suppression tumors were reduced in comparison with the negative control mice. (c) miR-657 expression levels in the different tumors were confirmed by RT-qPCR. (d and e) Overexpression of miR-657 promoted cell growth and suppressed apoptosis, while blocking of miR-657 expression reduced cell growth and induced cell apoptosis *in vivo*. (f) Western blot results also showed overexpression of miR-657 suppressed SRCIN1 and E-cadherin levels and enhanced N-cadherin, Vimentin, Slug, and Snail expressions in xenograft tumors.

Lastly, the role of miR-657 in NSCLC tumor growth was investigated in an *in vivo* xenograft model. A549 cells transfected with the miR-657 mimics or inhibitor were injected subcutaneously into the left flanks of the athymic nude mice, and the xenografts were injected with micrON hsa-miR-657 agomir or micrOFF hsa-miR-657 antagomir twice a week. It was found that, in agreement with the results of the cell experiments, the miR-657 overexpression tumors were significantly larger than those of control mice, while the miR-657 suppression tumors were reduced in comparison with the negative control mice (Figures 8(a) and 8(b)). miR-657 expression levels in the different tumors were confirmed by RT-qPCR (Figure 8(c)). Overexpression of miR-657 promoted cell growth and suppressed apoptosis, while blocking of miR-657 expression reduced cell growth and induced cell apoptosis *in vivo* (Figures 8(d) and 8(e)). Furthermore, Western blot results also showed that overexpression of miR-657 suppressed SRCIN1 and E-cadherin levels and enhanced N-cadherin, Vimentin, Slug, and Snail expressions in xenograft tumors (Figure 8(f)). All the above results suggested that miR-657 promoted cell growth and EMT *in vitro* and *in vivo*.

## 4. Discussion

The high rates of NSCLC incidence and associated morbidity and mortality post a major threat to public health throughout the world [2]. From a histopathological perspective, NSCLC is a heterogenous disease classification that can be separated into large cell carcinoma, squamous cell carcinoma, and adenocarcinoma subset accounting for 30%, 15%, and 40% of lung cancer patients, respectively [19, 20]. While NSCLC rates have been gradually declining with new advances in the treatment and diagnosis of this disease in its early stages, affected patients nonetheless exhibit relatively low 5-year survival rates [21]. High rates of metastasis are the primary drivers of treatment failure and poor outcomes in NSCLC patients.

As regulators of posttranscriptional target mRNA expression through binding to target 3′-UTR sequences [6], miRNAs play important roles in NSCLC diagnosis, treatment, and therapeutic resistance [22, 23]. Those miRNAs that exhibit NSCLC-specific expression patterns have the potential to be leveraged for the sensitive and specific diagnosis of this cancer type [24]. miR-503 was found downregulated in NSCLC tissues, and overexpression of miR-503 suppressed NSCLC cell multiplication [25, 26]. miR-510 is a potential oncogene in NSCLC through regulating SRCIN1 [27] and PTEN [28]. Furthermore, miR-647 promoted sensitivity to cisplatin and argon-helium cryoablation in NSCLC [29, 30]. Zhang et al. [11] previously ascertained that miR-657 was capable to promote malignant activity in HCC cells via targeting transducin-like enhancer protein 1 and modulating associated NF-*κ*B signaling. Moreover, miR-657 was found to be a potential biomarker to predict recurrence of localized stage I non-small-cell lung cancer after surgical resection [12]. Here, we discovered miR-657 to be upregulated in NSCLC cell lines and tissues. Overexpressing miR-657 markedly enhanced proliferation and invasiveness for both A549 and NCI-H1650 cells. Several miRNAs associated with the induction of the EMT have been linked to NSCLC progression, such as miR-135a, miR-134, miR-149, and miR-23a [31–34]. Our results suggest that overexpressing miR-657 was sufficient to upregulate Slug, Vimentin, and N-cadherin while promoting the downregulation of E-cadherin in both NCI-H1650 and A549 cells, suggesting that this miRNA can function as an oncogene to promote EMT induction in NSCLC cells.

SRCIN1 functions as a key mediator of Src inactivation, thereby suppressing the growth and development of a range of tumor types [35, 36]. In one report, for instance, Cao et al. [37] determined that miR-150 was able to suppress SRCIN1 translation in lung cancer, thereby functioning in an oncogenic manner, while Sharma et al. [38] revealed that SRCIN1 was capable to suppress tumor cell growth and invasion via suppressing Src or E-cadherin/EGFR signaling. In breast cancer, SRCIN1 expression was also found to be negatively associated with tumor malignancy, with Damiano et al. [35] demonstrating that STCIN1 was able to suppress the invasive activity of substantially metastatic breast carcinoma cells owing to its ability to suppress cortactin-dependent cell motility. Furthermore, suppression of SRCIN1 promoted cell multiplication, migration, and epithelial to mesenchymal transition (EMT) in pancreatic cancer cells [39]. Zhu and Han found that SRCIN1 is a target gene of miR-150-5p and overexpression of SRCIN1 suppressed cell proliferation and EMT in cervical cancer cells [40]. Moreover, miR-17-5p promoted cell multiplication and EMT in osteosarcoma (OS) through targeting SRCIN1 [41]. The studies indicated that SRCIN1 played critical roles in EMT. However, the specific functional role of SRCIN1 in NSCLC, however, has not been previously reported.

Here, it was explored that expression levels of SRCIN1 were reduced in NSCLC tumor tissues compared with paracancerous tissue specimens. We then explored the functional importance of SRCIN1 in the NCI-H1650 and A549 cells, revealing that its overexpression suppressed EMT induction in these cell lines. Ectopic SRCIN1 overexpression inhibited the proliferative and invasive activity of NSCLC cell lines, thus suggesting that this gene functions as a tumor suppressor gene in this oncogenic context. Bioinformatics analyses demonstrated that miR-657 was able to bind to SRCIN1 and to suppress its expression in both tested NSCLC cell lines. Importantly, SRCIN1 was capable to partially reverse the impact of miR-657 expression on the malignant activity of these NCI-H1650 and A549 cells. Together, these findings thus indicated that miR-657 is able to drive NSCLC cell invasiveness, proliferation, and EMT induction by suppressing SRCIN1 expression and modulating the Slug pathway within these tumor cells *in vitro*.

## Figures and Tables

**Table 1:** Correlation between miR-657 expression and clinicopathological features in NSCLC patients.

Parameters	Group	*N*	Expression of miR-657		*P* value
			Low, *n* (%)	High, *n* (%)	
Age (years)	≤58	46	22 (48.83)	24 (52.17)	0.6828
	>58	50	26 (52.00)	24 (48.00)	
Gender	Female	48	26 (54.17)	22 (45.83)	0.8374
	Male	48	27 (56.25)	21 (43.75)	
Tumor size (cm)	<3	40	17 (66.67)	23 (33.33)	0.2847
	≥3	56	30 (34.38)	26 (65.62)	
TNM stage	I–II	52	38 (73.08)	14 (26.92)	0.0003
	III–IV	44	16 (36.36)	28 (63.64)	
Smoking history	Yes	46	27 (58.70)	19 (41.30)	0.8966
	No	50	30 (60.00)	20 (40.00)	
Lymph node metastasis	Yes	42	14 (33.33)	28 (66.67)	0.0040
	No	54	34 (62.96)	20 (37.04)	
Differentiation	Well/moderate	47	32 (68.09)	15 (31.91)	0.0021
	Poor	49	18 (36.73)	31 (63.27)	
Subtypes	Squamous carcinoma	27	12 (44.44)	15 (55.56)	0.2536
	Adenomatous carcinoma	21	14 (66.67)	7 (33.33)	
	Adenosquamous carcinoma	26	10 (38.46)	16 (61.54)	
	Magnocellular carcinoma	22	10 (45.45)	12 (54.55)	

## Data Availability

The datasets used in this study are available from the corresponding author upon reasonable request.
